# Can a brief training intervention help improve mental health service delivery in South Africa?

**DOI:** 10.4102/phcfm.v13i1.2909

**Published:** 2021-10-26

**Authors:** Frances B. Slaven, Yvonne Erasmus, Margot Uys, Pierre-Emile Bruand, Beki Magazi, Rabia Wadvalla

**Affiliations:** 1 Department of Education Innovation, Faculty of Academic Cluster, Foundation for Professional Development, Pretoria, South Africa; 2 Africa Centre for Evidence, Faculty of Humanities, University of Johannesburg, Johannesburg, South Africa; 3 School of Health Sciences, Faculty of Academic Cluster, Foundation for Professional Development, Pretoria, South Africa; 4 Global Health, SANOFI, Paris, France; 5 Department of General Medicine, SANOFI, Johannesburg, South Africa; 6 Department of Psychiatry, Faculty of Health Sciences, University of KwaZulu-Natal, Durban, South Africa

**Keywords:** primary care, mental health, capacity development, integration of mental health care, programme evaluation

## Abstract

**Background:**

South Africa faces a number of significant challenges apropos mental health service delivery, including a large treatment gap, a high rate of readmission, over-burdened specialist tertiary facilities, and slow integration of mental health into general health services. The South African National Mental Health Education Programme implemented between February 2019 and December 2019, aimed to upskill health workers to diagnose and manage mental disorders at primary and secondary levels of care.

**Aim:**

This study aimed to assess the evolution of training participants’ self-reported competency in mental health care and the number of referrals made to higher levels of care as well as to reflect on the possible broader effects of the training.

**Setting:**

The programme and study were conducted in South Africa with Medical Officers and Professional Nurses working at public sector primary and secondary level health care facilities.

**Methods:**

A descriptive observational study collected data from training participants through a pre- and post-course, and 3-month follow-up survey.

**Results:**

The average confidence ratings for performing mental health care activities and managing mental health conditions increased from pre- to post-course, and was either maintained or increased further at 3-month follow-up. A decrease in the self-reported percentage of patients being referred to a higher level of care was observed 3-months after the training.

**Conclusion:**

The evaluation suggests that a brief training intervention such this can go a long way in increasing the confidence of primary and secondary level health care workers in managing common mental health conditions and adhering to the provisions of legislation.

## Introduction

The high burden of mental disorders in South Africa is well-established. The South African Stress and Health Study found that the 12-month prevalence of any Diagnostic and Statistical Manual of Mental Disorders, fourth edition (DSM-IV) defined disorder was 16.5%, whilst the lifetime prevalence for any mental disorder was found to be 30.3% with the most common disorders being anxiety-related disorders, major depressive disorder, and alcohol abuse.^1,2,3^ Despite this large burden, a low number of South Africans access mental health care services, a phenomenon known as the treatment gap.^4,5,6^ It is estimated that only 27% of South Africans with severe mental disorders receive treatment.^7^

South African mental health patients also experience high rates of re-admission to tertiary mental health institutions, a phenomenon known as the revolving door syndrome. In a systematic review, Lund and Peterson found that this is mainly because poor treatment adherence and defaulting; substance abuse; and early discharge because of bed shortages.^8^ Poor integration of mental health services into the general health services environment overwhelms the tertiary mental health care system and may contribute to the observed revolving-door phenomenon. The *Mental Health Care Act* (Act no. 17 of 2002) and the National Mental Health Policy Frameworks and Strategic Plan 2013–2020 provide for a shift towards decentralised and community based mental health care.^9^ The *Mental Health Care Act* prescribes that mental health should be integrated into general health services at primary, secondary and tertiary levels to improve access to mental health services and reduce the stigma associated with mental illness.^10^ This would require that primary and secondary health facilities have appropriately trained staff, facilities and access to medication, a reality that is still a work in progress.^11^

The *Mental Health Care Act* also sets out strict procedures that need to be followed by health care workers in the admission of mentally ill patients, especially in cases of assisted and involuntary care. Szabo and Kaliski noted that the administrative burden placed on health care workers has increased and they need to complete a ‘host’ of documents throughout the admission process to ensure that all decisions that are made are well thought through and defensible in court.^11^

To support countries in the shift towards decentralised care, the World Health Organization (WHO) identified 10 key principals for mental health integration, one of which was the adequate training of primary care workers in the diagnosis and treatment of mental health conditions.^12^ Research into the outcomes and effectiveness of short mental health training interventions for non-specialist health care workers have found significant improvements in knowledge, confidence, attitude towards mental health, clinical practice and skills, and patient outcomes.^13,14,15,16^ A study on a 5-day training intervention for primary health care workers in Ethiopia (medical officers [MO] and nurses) on the management of mental disorders found significant improvements in participants’ knowledge (42% average increase), attitude (43% average increase) and practice (45% average increase) after the training.^14^ Fewer studies have evaluated the effects of training on patient outcomes. A study conducted in Kenya found no significant improvement in the detection of mental health problems, but did find significant improvements in patient health, disability, and quality of life measures, 12-weeks after the training intervention.^17^ A systematic review of literature on interventions to improve provider recognition and management of mental disorders in primary care, found improved diagnosis of mental disorders reported in 18 of 23 studies examining this outcome, improved treatment in 14 of 20 studies, and clinical improvement in psychiatric symptoms in 4 of 11 studies.^18^

The South African National Mental Health Education Programme (SANMHEP) was a National Department of Health (NDoH) initiative to strengthen mental health services in South Africa in collaboration with the Foundation for Professional Development (FPD), SANOFI and the various provincial departments of health. The training programme was delivered using a blended learning approach consisting of a face-to-face workshop and e-learning.

Since the implementation of this programme, the coronavirus disease 2019 (COVID-19) pandemic has taken hold of the world. Initial data are suggesting that the incidence of mental disorders is increasing and that during the pandemic the condition of many people with pre-existing mental disorders is worsening. Social distancing, quarantine, lockdown and other restrictions implemented, as well as their subsequent economic and social consequences are leading to an increase in the burden of mental disorders.^19^ It is important, now more than ever, to equip health care workers with the tools needed to respond to this threat.

## Methods

### Study design

A descriptive observational outcome evaluation was designed to determine if the intervention had an influence on training participants’ self-reported competency and referral behaviour. The objectives of the evaluation were to: (1) determine if there was an increase in training participants’ self-reported competency in mental health care immediately after the course and 3 months later; (2) determine if there was a decrease in the number of self-reported mental health referrals made to higher levels of care; (3) describe the challenges faced by the training participants in providing mental health care once they were back at work; (4) describe the training participants’ overall experience of the training and how it has affected their work; (5) describe the training participants’ reflections on the possible broader, long-term effects of the training; and (6) describe how the course can be improved. For the purposes of this research, findings related to objectives 1, 2, 4 and 5 will be discussed. The other objectives of the evaluation may be reported on in future research.

### Study setting

The evaluation was conducted within the context of the training programme. Workshops were conducted in training venues across the country, including venues at Regional Training Centres and public health facilities, from 20 February 2019 to 29 November 2019.

### Study population

The training programme specifically targeted MO and professional nurses (PN) working within public facilities that are listed to conduct 72-h assessments (primary health care [PHC] clinics and psychiatric units attached to district and regional hospitals). The MOs and PNs from Correctional Service Centres and university PHC clinics were also included in the training.

### Sample size and selection of participants

There was no sampling of participants for the first part of the evaluation (pre- and post-workshop survey) as all trainees were asked to complete a questionnaire before and after the workshop. A requirement to be included in the second part of the evaluation (the follow-up survey) was to complete the online component of the training. All trainees who completed the online component of the training were invited to complete the follow-up survey.

### Intervention

The SANMHEP programme aimed to upskill MOs and PNs to diagnose mental disorders and provide quality care, treatment and rehabilitation within the facility. It also aimed to streamline referral indications and processes so that only those mental health care users who require specialised services are referred. The programme was implemented through a short course using a blended-learning approach consisting of a 3-day face-to-face workshop, provided by psychiatrists and mental health practitioners with significant experience in the field, and an online component delivered by the FPD. Students had to complete both components to be awarded with a certificate of completion. The course covered treatment guidelines for common mental disorders; conducting psychiatric interviews and conducting mental health screening, assessment and treatment; the *Mental Health Care Act*; referral pathways; as well as guidelines, flowcharts, and other resources.

A total of 1120 health care workers, the majority of which were PNs and medical doctors, were trained through 36 workshops between February and November 2019. Workshops took place across all nine provinces of South Africa, with Gauteng having the highest number of students, followed by KwaZulu-Natal and Mpumalanga. The majority of students were female (69%) and worked in a district hospital (53.4%).

Approximately one-third of the workshop attendees (337; 30%) completed the online component of the training that was required to receive a certificate.

### Data collection

Data were collected from training participants through a pre- and post-course survey which participants completed before and after the workshop, and a 3-month follow-up survey. Only those students who completed the online component of the training were invited to complete the follow-up survey. The pre-course, post-course and follow-up survey asked the training participants to rate their competency in performing 11 mental health care activities and in managing 30 mental health conditions, to estimate the percentage of mental health patients they refer to higher levels of care, and to describe the mental health care resources available to them, as well as challenges faced in providing mental health care.

### Data analysis

Only those students who had completed all three surveys were included in the analysis. Quantitative data were coded and initially descriptively analysed in MS Excel^®^. Data from the three surveys were compared to determine if there was an increase in training participants’ self-reported competency in mental health care, and if there was a decrease in the number of self-reported mental health referrals made to higher levels of care immediately after the course and 3 months later. Among the participants who completed the pre-, post-, and follow-up course assessment, one-way repeated measures analysis of variance (ANOVA) was performed with STATA 13.1 software (Stata Corp, College Station, Texas, United States [US]) to determine if there was a statistically significant difference in the confidence rating of performing mental health activities and managing mental health activities from the pre-course assessment.

### Ethical considerations

Research ethics approval for this evaluation was obtained from the Foundation for Professional Development’s Research Ethics Committee (registration number: REC-03711-033-RA Level 1) in October 2019.

## Results

Of the 337 students invited to participate in the follow-up survey, 53 responded (15.7% response rate).

The majority of the 53 participants were female (75.5%), PNs (71.7%), working in a district hospital (45.3%). Eight of the nine South African provinces were represented in the final sample with 16% from the Free State and Mpumalanga, respectively, 14% from the Eastern Cape and Gauteng, respectively, 13% from KwaZulu-Natal, 11% from Limpopo, 7% from the Western Cape, and 4% from the North-West.

Competency ratings for the 11 mental health care activities and 30 mental health conditions were from 1 to 10 with 1 being not at all confident and 10 being extremely confident.

The overall average confidence rating for performing mental health care activities increased significantly from 5.8 before the course to 8.2 immediately after the course, and was maintained at 8.2 at 3-month follow-up (*p* < 0.001) (Table 1). The confidence ratings of all activities significantly increased from the pre-course assessment (Table 1). The activities with the highest post-course and follow-up ratings were: conducting a psychiatric interview; conducting a mental status examination; post-test human immunodeficiency virus (HIV) counselling; and placing a patient under involuntary care and treatment. The biggest increase from pre-course to 3-month follow-up was observed for the activity of placing a patient under involuntary care and treatment (pre: 5.7, follow-up: 8.8).

**TABLE 1:** Pre-course, post-course, and follow-up survey competency ratings for performing mental health care related activities.

Mental health care related activities	Average competency rating (*n* = 53)			
	Pre-course	Post-course	Follow-up	*p*
Conducting a psychiatric interview	6.3	8.7	8.6	< 0.001
Conducting a psychiatric evaluation	6.1	8.3	8.4	< 0.001
Conducting a mental status examination	6.3	8.5	8.7	< 0.001
Conducting blood investigations in mental health	5.6	8.0	8.2	< 0.001
Conducting drug/substance screening	5.8	7.8	8.1	< 0.001
Classifying psychiatric conditions with the DSM-5	5.0	7.8	7.5	< 0.001
Psychiatric rating scales (e.g. Brief Psychiatric Rating Scale	4.4	8.1	7.2	< 0.001
Post-test HIV counselling	7.3	8.1	9.0	< 0.001
Prescribing psychiatric medications	4.4	7.7	7.4	< 0.001
Emergency treatment without consent	5.3	8.4	8.3	< 0.001
Placing a patient under involuntary care and treatment	5.7	8.7	8.8	< 0.001

**Overall average**	**5.6**	**8.2**	**8.2**	**< 0.001**

DSM-5, Diagnostic and Statistical Manual of Mental Disorders, fifth edition; HIV, human immunodeficiency virus.

The overall average confidence rating for managing mental health conditions increased significantly from 5.8 before the course, to 7.6 immediately after the course, and to 7.9 at 3-month follow-up (*p* < 0.001) (Table 2). The confidence ratings for all mental health conditions significantly increased from the pre-course assessment except for managing disturbances in intelligence and feeding/eating disorders (Table 2). The conditions with the highest post-course and follow-up confidence ratings were depressive disorders, schizophrenic disorders, psychotic disorders, and bipolar disorders. The biggest increase from pre-course to follow-up was for dissociative disorders, psychiatric emergencies, and violent patients.

**TABLE 2:** Pre-course, post-course, and follow-up survey competency ratings for managing mental health conditions.

Mental health conditions	Average competency rating (*n* = 53)			
	Pre-course	Post-course	Follow-up	*p*
Disturbances of consciousness	6.0	8.1	7.8	< 0.001
Disturbances of attention	5.8	7.9	7.9	< 0.001
Disturbances in suggestibility	5.3	7.3	7.3	< 0.001
Disturbances in motor behaviour	5.8	7.5	7.5	< 0.001
Disturbances in thinking	5.7	7.8	7.6	< 0.001
Disturbances in speech	5.8	7.6	7.8	< 0.001
Disturbances of perception	6.0	7.9	8.0	< 0.001
Disturbances of memory	6.1	7.6	8.1	< 0.001
Disturbances in intelligence	6.8	7.6	7.8	0.61
Neurocognitive disorders	5.2	7.5	7.5	< 0.001
Neurocognitive disorders secondary to HIV infection	5.6	7.2	7.8	< 0.001
Schizophrenic disorders	6.1	7.6	8.4	< 0.001
Psychotic disorders	6.3	7.7	8.4	< 0.001
Depressive disorders	6.2	7.9	8.5	< 0.001
Bipolar disorders	6.2	8.1	8.4	< 0.001
Anxiety disorders	6.4	7.9	8.3	< 0.001
Suicidal ideation/risk of suicide	6.3	7.9	8.3	< 0.001
Obsessive-compulsive disorders	6.1	7.6	8.1	< 0.001
Trauma and stress-related disorders	6.2	7.8	8.3	< 0.001
Personality disorders	5.3	7.4	7.5	< 0.001
Somatic symptom disorder	5.3	7.4	7.6	< 0.001
Functional neurological symptom disorder	5.0	6.9	7.1	< 0.001
Dissociative disorders	4.7	6.8	7.2	< 0.001
Disruptive, impulse-control and conduct disorders	5.2	7.0	7.4	< 0.001
Violent patients	5.7	7.9	8.1	< 0.001
Substance related and addictive disorders	5.9	7.9	8.2	< 0.001
Feeding and eating disorders	7.1	7.0	7.8	0.91
Psychiatric disorders in elderly people	5.9	7.5	7.9	< 0.001
Psychiatric disorders in children	4.8	7.1	7.1	< 0.001
Psychiatric emergencies	5.9	8.2	8.3	< 0.001

Overall average	5.8	7.6	7.9	< 0.001

HIV, human immunodeficiency virus.

**FIGURE 1:**
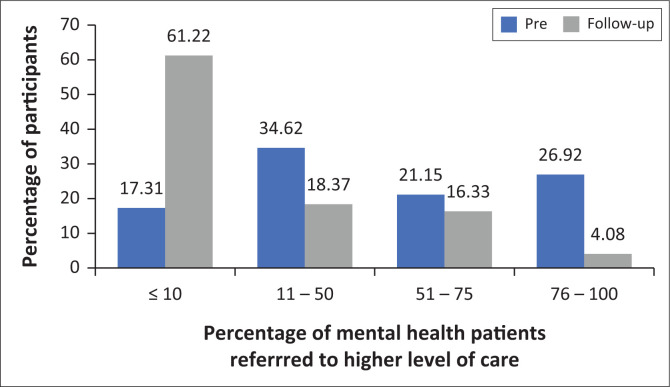
Estimated percentage of mental health patients being referred to a higher level of care pre-course and at follow-up (*n* = 53).

In the pre-course and follow-up survey, the participants were asked to select the percentage of mental health patients that they usually referred to higher levels of care with the options being: ≤ 10%; 11% – 50%; 51% – 75%; or 76% – 100%. Before the workshop, only 17.3% of the participants selected ≤ 10%. At 3-month follow-up 61% of the participants selected ≤ 10% (Figure 1) implying that health care professionals felt more confident and equipped to assess patients and provide appropriate treatment at lower entry points to the health care system.

## Discussion

### Key findings

The participants’ confidence in performing mental health care related activities and in managing mental health conditions increased immediately after the course and increased at follow-up. The analysis of variance showed that the pre-course and follow-up overall average ratings for performing activities and managing mental health conditions were significantly different from one another.

The percentage of participants who reported that they referred less than 10.0% of their mental health patients to higher levels of care increased by 43.7%.

### Discussion of key findings

The findings suggest that the training programme increased participants’ confidence in their ability to manage mental health conditions in their facility. The increases in confidence observed at follow-up may be because of the students putting what they have learnt into practice.

The self-reported decrease in the percentage of mental health care patients referred to higher levels of care found at follow-up provides an indication of the possible medium-term effects of the training programme.

The ability to manage and monitor mental health care patients’ conditions at primary care level may reduce the risk of relapse and subsequent need for re-admission, thereby contributing to the easing of the revolving door syndrome.

### Limitations

The low number of students who completed both the workshop and the online components of the course resulted in a small sample size for the evaluation. The low response rate to the follow-up survey further reduced the sample for the statistical analyses. This selection bias may positively bias the results because students who are continually engaged in the course generally experience larger benefits from the course. The reasons for non-completion of the online component will be investigated in further studies.

### Implications and recommendations

Training on the management of common mental health conditions in primary and secondary care settings should be made available to more MOs and PNs as it has been shown to improve health care workers’ confidence and the number of referrals made to higher levels of care.

Several important lessons were learnt during the implementation of this programme which may be valuable to others who are implementing similar programmes. Using a blended learning approach was found to be an effective way of delivering this training as it minimised the number of days that participants would need to be away from the facility. However, the low completion rate of the e-learning component of the course highlighted the challenges of providing online learning to this population. It is recommended that courses such as this continue to use traditional modes of delivery (e.g. workshops), supplemented by online engagement. Another solution to increase completion rates may be to require participants to complete the online component of the training before attending the workshops. This will also enable students to work through the course material before the workshop, allowing the facilitator to solely focus on application and areas that need clarification.

## Conclusion

The evaluation provided evidence to show that a brief training intervention such as a short course can go a long way in increasing the capacity and confidence of primary and secondary level health care workers in managing common mental health conditions and adhering to the provisions of the *Mental Health Care Act*.

These outcomes may lead to broader system level improvements including a decrease in the burden placed on tertiary mental health care hospitals and an improvement in the revolving door syndrome. Furthermore, treating patients closer to where they live will save them and their families the time and costs of travelling to further away tertiary facilities.

Further research on the impact of similar training interventions needs to be conducted including determining impact on the number of admissions to tertiary facilities and the revolving door syndrome. Other areas of future research could include a cost-benefit analysis to determine if the medium- and long-term outcomes of the intervention justify the resources needed to implement it and a compliance audit to determine the extent to which the provisions of the *Mental Health Care Act* are adhered to.
